# Risk factors for catheter-related bloodstream infections in a surgical ICU

**DOI:** 10.1186/cc11987

**Published:** 2013-03-19

**Authors:** A Kundakci, O Ozkalayci, P Zeyneloglu, H Arslan, A Pirat

**Affiliations:** 1 Baskent University Hospital, Ankara, Turkey

## Introduction

Preventing catheter-related bloodstream infections (CR-BSI) can reduce the duration of hospital stay, healthcare costs, and mortality rates. Identifying the risk factors and correction of modifiable factors should be one of the main objectives of infection control measures. The aim of this study is to determine the risk factors of CR-BSI in a cohort of surgical ICU patients admitted to Baskent University Hospital.

## Methods

Following Institutional Review Board approval, data for 876 patients admitted to the surgical ICU between January 2009 and July 2012 were reviewed retrospectively. After completing the review, 25 patients diagnosed with CR-BSI were compared with 66 appropriate matches who did not have CR-BSI. Demographical features, underlying diseases, APACHE II (Acute Physiology and Chronic Health Evaluation) and SOFA (Sequential Organ Failure Assessment) scores, length of stay, organ dysfunctions, laboratory values, use of vasopressors, mechanical ventilation, nutrition, antibiotics, transfusions, and features related to central venous catheterization were recorded. Patients who did not have a central venous catheter and were discharged within 2 days of ICU stay were excluded.

## Results

Out of 91 patients included in the final analysis, 25 (27%) patients had CR-BSI. When compared with patients who did not have CR-BSI, those who did were older (*P *= 0.029), required more blood product transfusions during the first 3 days of ICU (*P *= 0.016), had a longer duration of catheter stay (*P *= 0.019), and were more frequently catheterized via the internal jugular vein (IJV) (*P *= 0.022). A logistic regression model revealed that advanced age (OR: 1.037; 95% CI: 1.001 to 1.073; *P *= 0.042) was an independent risk factor for CR-BSI. Fourteen-day and 28-day mortality rates for CR-BSI were 12% (*P *= 0.749) and 28% (*P *= 0.406), respectively.

## Conclusion

Although age, blood product transfusion, duration of catheter stay, and use of IJV were different between patients who did and did not have CRBSI, advanced age was the only independent risk factor for CR-BSI. Early suspicion of CR-BSI by the other well-known risk factors has a substantial effect on the treatment of CR-BSI.
